# Impairment of Executive Functions Associated With Lower D-Serine Serum Levels in Patients With Schizophrenia

**DOI:** 10.3389/fpsyt.2021.514579

**Published:** 2021-03-29

**Authors:** Jaromir Hons, Rastislav Zirko, Martina Vasatova, Pavel Doubek, Blanka Klimova, Jiri Masopust, Martin Valis, Kamil Kuca

**Affiliations:** ^1^Center for Psychiatry, Regional Hospital Liberec, Liberec, Czechia; ^2^Institute of Health Studies, Technical University of Liberec, Liberec, Czechia; ^3^Department of Psychiatry, Faculty of Medicine in Hradec Kralove, University Hospital Hradec Kralove and Charles University in Prague, Hradec Kralove, Czechia; ^4^Institute of Clinical Biochemistry and Diagnostics, University Hospital Hradec Kralove and Charles University in Prague, Hradec Kralove, Czechia; ^5^Department of Psychiatry, 1st Faculty of Medicine, General Teaching Hospital and Charles University in Prague, Prague, Czechia; ^6^Department of Neurology, Faculty of Medicine in Hradec Kralove, University Hospital Hradec Kralove and Charles University in Prague, Hradec Kralove, Czechia; ^7^Biomedical Research Center, University Hospital Hradec Kralove, Hradec Kralove, Czechia; ^8^Faculty of Science, University of Hradec Kralove, Hradec Kralove, Czechia

**Keywords:** executive functions, D-serine, schizophrenia, excitatory amino acids, dysregulation of glutamatergic neurotransmission

## Abstract

A core symptom that is frequently linked with dysregulation of glutamatergic neurotransmission in regard to schizophrenia is impairment or damage of executive functioning as a component of cognitive deficiency. The amino acid D-serine plays the role of an endogenous coagonist at the glutamatergic *N*-methyl-D-aspartate (NMDA) receptor glycine modulatory site. Considerably reduced serum levels of D-serine were found in patients suffering from schizophrenia compared with healthy control participants. An increase in D-serine led to augmented cognitive functionality in patients suffering from schizophrenia who were undergoing clinical trials and given the treatment of first- and second-generation antipsychotics. The study proposed the hypothesis that the D-serine blood serum levels may be linked with the extent of executive functionality in those suffering from the mental illness in question. For the purpose of examining executive function in such patients, the Rey–Osterrieth Complex Figure, Trail Making, and Wisconsin Card Sorting tests were applied (*n* = 50). High-performance liquid chromatography was used to gauge the total serine and D-serine levels. The extent of damage was examined through neuropsychological tests and was found to be considerably linked to D-serine serum level and the D-serine/total serine ratio (*p* < 0.05) in the sample being considered. A lower average serum level of D-serine and lower D-serine/total serine ratio were observed in participants with the worst performance compared with those displaying the best performance—this was true when the patients were split into quartile groups based on their results (*p* < 0.05). The findings of modified D-serine serum levels and the D-serine/total serine ratio linked to the extent of damage in executive functioning indicate that serine metabolism that is coresponsible for NMDA receptor dysfunction has been changed.

## Introduction

Clinically significant impairment of cognitive functions occurs in 40–60% of people suffering from schizophrenia (1). The term “cognitive” relates to a wide range of mental and intellectual abilities that depend on the function of the cerebral cortex. Disturbances in attention, memory, executive functions, and certain aspects of speech and language are repeatedly found on average as reduced performance in neuropsychological testing in the patients. Approximately only 15% of patients in the remission phase of schizophrenia exhibit cognitive performance levels comparable with those of healthy people (2). Cognitive impairments are of crucial functional importance in patients with schizophrenia.

Impairment in executive functioning when taken in the context of cognitive impairment is typically highlighted as a core symptom linked to glutamatergic neurotransmission dysregulation in schizophrenia (3). The competitive *N*-methyl-d-aspartate (NMDA) receptor antagonists ketamine and phencyclidine have been consistently reported to provoke psychopathology similar to schizophrenia in human subjects, including cognitive symptoms (3–7). The results from treatment trials that augmented glutamatergic neurotransmission basically show that NMDA receptors modulated with the help of excitatory amino acids (EAAs), such as glycine or d-serine, play a role when used as adjunctive treatments to address the symptoms in question. The NMDA receptor coagonists glycine (8–10) and d-serine (11, 12) improved cognitive functionality for patients suffering from the disease in question. This is true when they are applied to enhance treatment alongside the use of antipsychotic drugs (Table 1). The results used data from 26 studies to perform the meta-analysis, highlighting evidence to confirm that multiple schizophrenia symptoms saw improvement, including cognitive symptoms, when d-serine or glycine was used (16). The two showed efficiency when used in patients with the disease who were under treatment with olanzapine or risperidone but were not given clozapine (16). Despite this, a systematic review of 18 short-duration trials covered under the meta-analysis demonstrated that the impact of the two i.e., d-serine and glycine, were not noticeable in terms of cognitive deficits, but they improved negative symptoms (17).

**Table 1:** D-Serine and glycine in augmentation of treatment with antipsychotics in patients with schizophrenia—results of the clinical trials.

**References**	***N* (P/A)**	**Method duration**	**Administration**	**Daily dose**	**Ongoing antipsychotics**	**Improved symptoms**
Heresco-Levy et al. (9)	11	DB, PC 6 weeks	Glycine	0.8 g/kg	FGA/SGA	Negative symptoms Depressive symptoms Cognitive functions
Tsai et al. (11)	15/14	DB, PC 6 weeks	D-Serine	30 mg/kg	FGA/SGA	Positive symptoms Negative symptoms Cognitive functions
Javitt et al. (8)	12	DB, PC 6 weeks	Glycine	60 g	FGA/SGA	Negative symptoms Cognitive functions
Heresco-Levy et al. (10)	17	DB, PC 6 weeks	Glycine	0.8 g/kg	Olanzapine or risperidone	Positive symptoms Negative symptoms Cognitive functions
Heresco-Levy et al. (12)	38	DB, PC 6 weeks	D-Serine	30 mg/kg	Olanzapine or risperidone	Positive, negative, and depressive symptoms, cognitive functions
Kantrowitz et al. (13)	42	OL 4 weeks	D-Serine	30 mg/kg 60 mg/kg 120 mg/kg	FGA/SGA	Positive symptoms Negative symptoms Cognitive functions
D'Souza et al. (14)	104	DB, PC 12 weeks	D-Serine	30 mg/kg	FGA/SGA	—
Kantrowitz et al. (15)	16	DB, PC 6 weeks	D-Serine	60 mg/kg	FGA/SGA	Positive symptoms Negative symptoms Cognitive functions MMN generation

*N, number of patients who completed the trial; P/A, placebo/augmentation subjects; DB, double-blind; PC, placebo-controlled; OL, open-label; FGA, first-generation antipsychotics; SGA, second-generation antipsychotics; MMN, mismatch negativity*.

Augmentation by glycine improved cognitive symptoms (8–10) in patients treated with first-generation antipsychotics (FGAs) or second-generation antipsychotics (SGAs). In contrast, treatment with clozapine, when augmented by glycine, did not show any treatment benefits (18, 19). Buchanan also found no effect of augmentation by glycine on cognitive symptoms compared with a placebo in the largest treatment study in patients receiving FGAs or SGAs (20). Peroral therapeutic administration of EAAs caused changes in the EAA serum levels in clinical trials. Adjunctive treatment with glycine enhanced serum glycine levels (8, 9). Higher glycine serum levels after treatment with glycine were linked to an improved clinical effect (10). Augmentation by sarcosine (*N*-methylglycine), a glycine transporter I inhibitor, also appeared effective on cognitive symptoms (21, 22) in patients with schizophrenia treated with antipsychotics. In agreement with the results of adjunctive treatment with glycine (18, 19), augmentation of clozapine treatment by sarcosine did not appear to be effective (23).

Amino acid d-serine is an endogenous selective full coagonist at the glycine modulatory site of the NMDA receptor and acts as a modulator of glutamatergic neurotransmission, neuronal migration, and long-term potentiation (LTP) (24). Decreased d-serine levels were found in the blood serum of patients with schizophrenia compared with healthy controls (25–27). Bendikov et al. (28) detected decreased d-serine levels and d/l-serine ratios in the cerebrospinal fluid (CSF) of patients with schizophrenia. A low ratio of d-serine to total serine was also found in the CSF of drug-naive first-episode patients with schizophrenia (29). Clinical studies have been conducted to evaluate body fluid levels of d-serine and to confirm its therapeutic effect as augmentation of treatment with antipsychotics in patients with schizophrenia (30). Augmentation by d-serine improved cognitive symptoms in patients treated with SGAs (11, 12). Positive therapeutic effects of d-serine not only on negative but also positive symptoms and dose-dependent effects on neurocognitive functions were confirmed in patients with schizophrenia (13). These results were supported by findings of improvement in mismatch negativity generation associated with clinical symptom improvement during d-serine treatment (15, 31). In contrast, studies with clozapine (32) and risperidone (21) did not detect any treatment effect of augmentation by d-serine. This has been tentatively explained by the partially agonistic effect of these antipsychotics at the NMDA receptor. Additionally, d-serine treatment in combination with computerized cognitive retraining did not show any significant effect on cognitive functions in patients with schizophrenia treated with antipsychotics in a placebo-controlled study (14). However, significantly increased d-serine levels correlated with improvement in global cognition and in verbal learning were observed after intensive cognitive training in patients with schizophrenia (27). The enhancement of d-serine serum levels was also observed after d-serine augmentation of the treatment with FGA or SGA (11). The total serine serum levels increased after augmentation by d-serine in patients treated with olanzapine or risperidone (12). The changes in EAA serum levels were not always associated with a positive clinical impact in patients treated with antipsychotics augmented with EAAs. However, the observed changes in EAA serum or plasma levels may reflect the improvement of the altered EAA metabolism and consequently the improvement of the clinical course.

Our study proposed the hypothesis that d-serine blood serum levels are linked to the level of executive functionality in people suffering from the disease. Our main aim was to determine whether these levels played a role when the patients had been given SGA or FGA treatment. We proposed that those with a higher degree of impairment in executive functioning would present with lower levels of d-serine serum, which is in line with the results of the changes of d-serine serum levels and treatment results on cognitive functioning in clinical trials that have been previously held (11, 12).

## Materials and Methods

### Study Population

Fifty patients 18 years or older were found from the outpatient clinic at the Department of Psychiatry, University Hospital Hradec Kralove, Czech Republic, in the study. The patients who were included in the sample were informed about the requirements and nature of the study being conducted. Fifty individually age- and sex-matched healthy subjects as controls with no history of psychiatric or neurological disorders or renal dysfunction and free of any psychotropic medication were also enrolled in the study. All participants provided consent before they were added to the study. The protocol being used for the study was examined and approved by the local ethics committee. The participants who were chosen were checked to ensure that they had no past record of substance use or abuse. For this purpose, they were checked for positive urine toxicology before they underwent the screening process. They were also checked for whether they had been subjected to any electroconvulsive treatment in the 5-month period prior to the study commencement. The women who were a part of the study had not been pregnant during this time. Two experienced psychiatrists helped provide the needed diagnoses under the *ICD-10* Diagnostic Criteria for Research (33). Paranoid schizophrenia was the most frequent diagnosis (*n* = 35), and the other schizophrenia types were residual (*n* = 7), undifferentiated (*n* = 4), hebephrenic (*n* = 2), and simplex (*n* = 1). A medical history of neurological disorders, cardiovascular disorders, or renal dysfunction reported by patients or documented in their records led to exclusion from the study population. The patients were physically healthy upon examination, and their laboratory assessments were within physiological limits. Four patients used FGA fluphenazine decanoate (25 mg/14–35 days; *n* = 3) and chlorpromazine (50 mg/d; *n* = 1) as monotherapy. SGA amisulpride (100–800 mg/d; *n* = 3), ziprasidone (80–160 mg/d; *n* = 3), long-acting risperidone (25–37.5 mg/14 d; *n* = 6), sertindole (12–24 mg/d; *n* = 2), olanzapine (5–20 mg/d; *n* = 9), quetiapine (600–1,400 mg/d; *n* = 3), clozapine (100–500 mg/d; *n* = 8), or aripiprazole (15 mg/d; *n* = 2) were administered at the time of the assessments in 36 patients as monotherapy. Five patients used a combination of FGA and SGA: haloperidol decanoate (50 mg/14–21 d) and risperidone (1.5–6 mg/d; *n* = 2), haloperidol (3 mg/d) and risperidone (6 mg/d; *n* = 1), haloperidol decanoate (50 mg/21 d) and olanzapine (35 mg/d; *n* = 1), and haloperidol (1.5 mg/d) and quetiapine (800 mg/d; *n* = 1). Four patients were administered a combination of two SGAs consisting of clozapine (150–450 mg/d) and aripiprazole (15 mg/d; *n* = 2) or long-acting risperidone (25–37.5 mg/14 d) and aripiprazole (15 mg/d; *n* = 2).

### Determination of Total Serine and D-Serine Serum Levels

Blood samples were collected after a 12-h period during which the subject was fasting. Here, the samples were used to determine the total and d-serine levels, and the sample was taken from the patient through venipuncture. The samples were taken between 8:00 and 9:30 am immediately before breakfast. This was done to avoid any EAA content–created bias because of food intake.

#### Sample Preparation

Venous blood was taken through a BD Vacutainer® SST^TM^ II Advance tube. It was then sent to the laboratory within the hour subsequent to extraction. The serum was centrifuged and deproteinated by ultrafiltration in Microcon columns (Millipore, USA). d-Norvaline was used as an internal standard. Clear filtrate was stored at −20°C until analysis.

#### Derivatization

The derivatization reagent contained 50 mg *o*-phthaldialdehyde in 5 mL methanol, 10 mL 0.2 mol/L boric acid in 0.2 mol/L potassium chloride, 10 mL 0.2 mol/L sodium hydroxide, and 2 mL 3-mercaptopropionic acid for total serine or 1.52 g *N*-acetyl-l-cysteine for d-serine determination (pH 9.3). The volume ratio of derivatization reagent to sample was 2:1, and the reaction time was 10 min (34–37).

#### Analysis

A gradient mode was used on the high-performance liquid chromatography to perform the chromatographic determination of total and d-serine. The system type was LC-10A vp (Shimadzu, Japan). The processes included three different phases, all of which were mobile. This included (A) 0.05 mol/L acetate buffer (pH 7.3), (B) 0.1 mol/L sodium acetate–acetonitrile–methanol (46:44:10), and (C) methanol (34, 36, 37). Chromatographic determination of d-serine was conducted in gradient mode and built around two main aspects i.e., eluent that had 0.21 mol/L sodium acetate that had been brought to pH 5.6 alongside the acetic acid concentrate and then methanol as the second eluent (38). The solution was separated through a Lichrocard 250x4 Lichrospher RP-18e column, which included 5-μm particles that were brought in from Germany *via* Merck. A fluorescence detector was used to find the signal (λem/λex 455/230 nm for total serine; λem/λex 454/337 nm for d-serine) (34, 36, 38). The average reproducibility was registered at 8.91% for total serine and 4.54% for d-serine. We found that the detection was limited at 2.58 μmol/L for total serine and 0.27 μmol/L for d-serine, and the bias values were 4.12% for total serine and 1.58% for d-serine.

### Clinical Assessments

Psychopathology evaluations were conducted in patients, and they were also administered a neuropsychological battery on the same day within the 4-h period after blood collection. The Positive and Negative Syndrome Scale (PANSS, 30 items, scores 1–7) (39) and the Scale for the Assessment of Negative Symptoms (SANS, 30 items, scores 0–5) (40) were employed for psychopathology assessments. A fully qualified and trained psychiatrist, with experience in rating (JH), performed all rating scales in the patients.

Neuropsychological tests evaluating different cognitive domains were grouped into batteries for testing executive functions, including the Trail Making Test (TMT; attention, speed of information processing, visual-motor coordination), Rey–Osterrieth Complex Figure Test (ROCFT; memory functions, learning), and Wisconsin Card Sorting Test (WCST; reasoning and problem solving), and administered by a fully qualified clinical psychologist (RZ). TMT is a well-established multiple susceptibility test in multiple cognitive domains. However, performance in TMT time: part B (TMT-B) differs from performance in TMT time: part A (TMT-A) in factors of motor control and perceptual complexity. The cognitive flexibility is captured in TMT-B performance mainly. Thus, the TMT-B/TMT-A ratio of performance in TMT was determined as a clearer index of executive functions (41).

### Statistical Analysis

**Figure 1:**
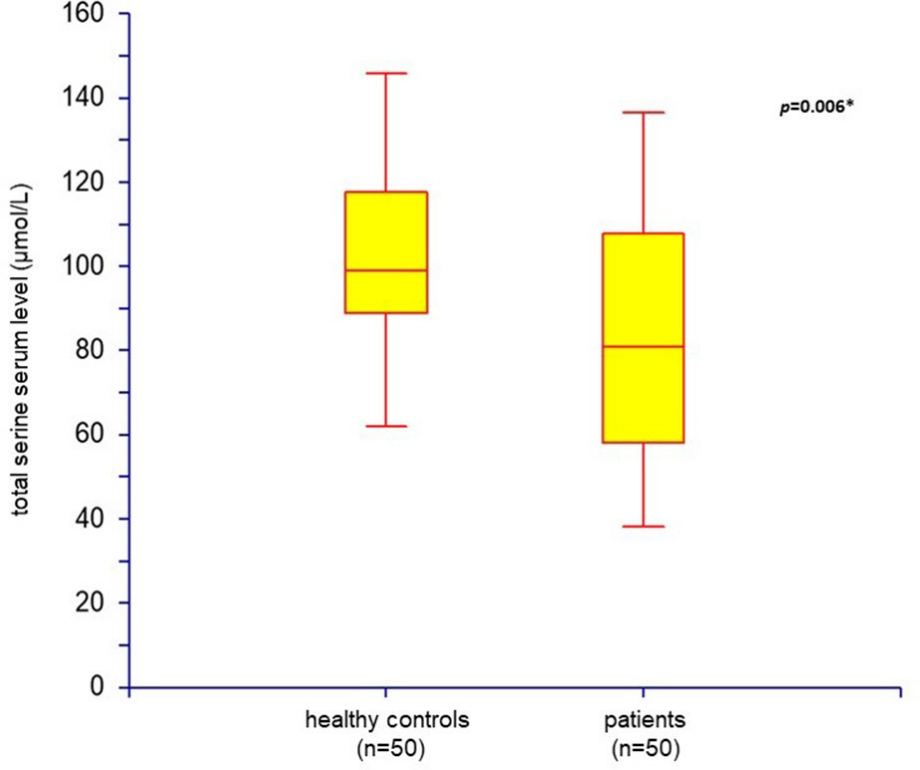
Total serine serum level: comparison of healthy controls (*n* = 50) and patients with schizophrenia (*n* = 50) (box plot; *p < 0.05 was considered as significant).

**Figure 2:**
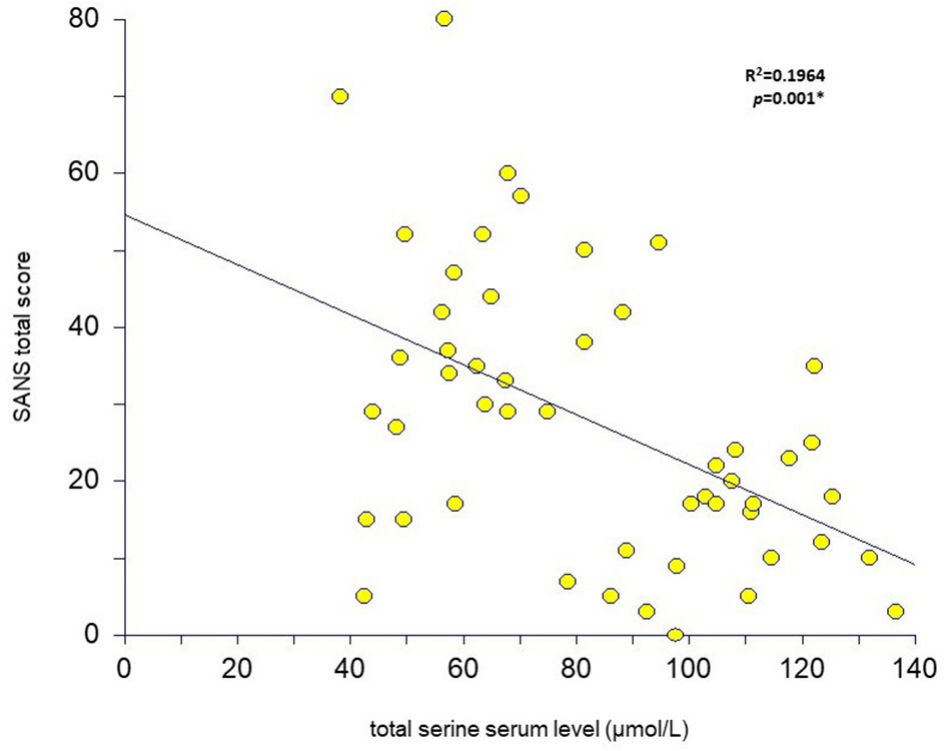
Total serine serum level relation to SANS total score in patients with schizophrenia (*n* = 50) (scatter plot with the linear regression curve; *p < 0.05 was considered as significant).

**Figure 3:**
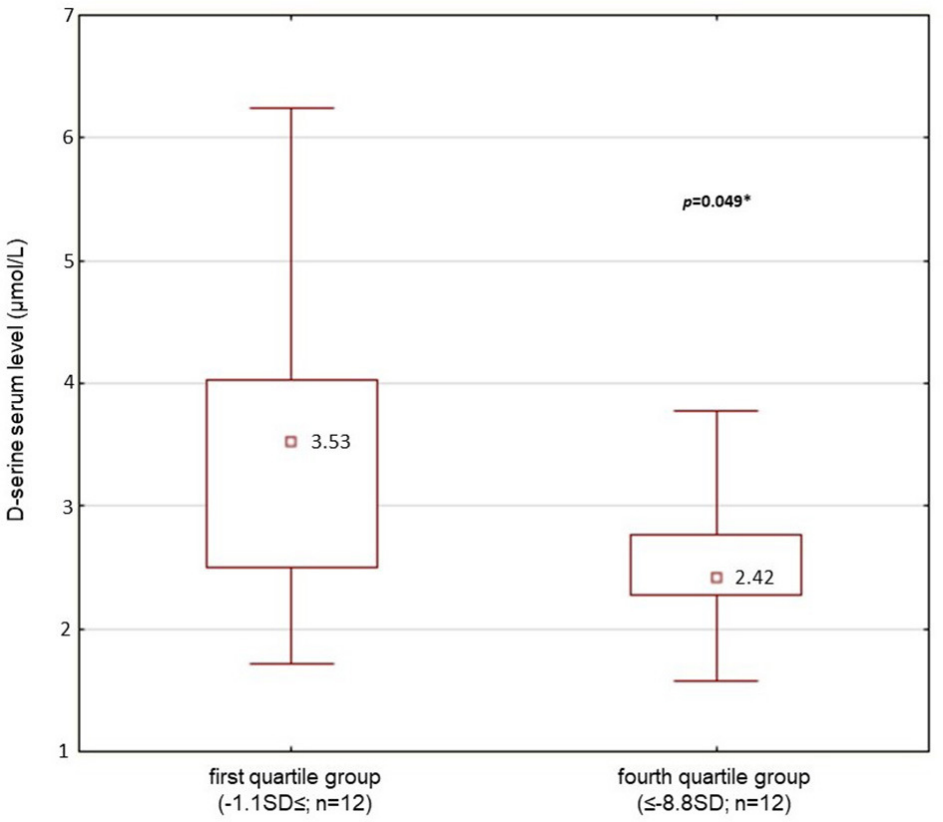
D-serine serum level (median; μmol/l): comparison of first quartile group (the best performance in executive function testing, the summary index 2; *n* = 12) and 4th quartile (the worst performance, the summary index 2; *n* = 12) of patients' population (*n* = 50) (Mann-Whitney *U*-test; *p < 0.05 was considered as significant).

Because of the lack of normality of the data distribution, non-parametric tests were used for mean comparisons. The Mann–Whitney *U*-test and Kolmogorov–Smirnov test were used for group comparisons of the average d-serine, total serine, and d-serine/total serine ratio values. Correlations of variables were computed by Spearman rank correlation (pairwise deletion, α = 0.05). Box plot and scatter plot were used for graphical presentation of the data (Figures 1, 2). The mean and SD of the normal control groups were used to transform the raw data from the results of particular neuropsychological tests in patients into *Z* scores for the TMT and ROCFT adapted from Mitrushina et al. (42) and the WCST adapted from Heaton et al. (43). The average *Z* scores from the results of the particular tests were calculated. Summary index 1 from the *Z* scores from TMT-B, ROCFT, and WCST as the degree of impairment in executive functioning was determined to enable testing the association of performance with biological variables. There are cognitive demands placed on the patient by TMT-B over and above those required to perform TMT-A, mainly cognitive flexibility and ability to maintain a complex response set (44). Thus, TMT-A average *Z* score was not included into summary index 1. Summary index 2 from the *Z* scores from ROCFT and WCST only was also determined. Spearman correlation coefficients (α = 0.05) were used to test the relationship between two parameters. The patients' population was divided into quartile groups according to their performance in the neuropsychological tests. The Mann–Whitney *U*-test was used to examine differences between groups. α = 0.05 was considered significant. Box plot was used for graphical presentation of the data (Figure 3).

## Results

Fifty outpatients with schizophrenia (33 males and 17 females) and 50 age- and sex-matched healthy controls were enrolled in the study. Serum assays of endogenous d-serine and total serine were performed in all subjects. The mean scores of demographic data, psychopathology assessments performed in patients, and the values of mean serum d-serine and total serine levels are listed in Table 2. The mean serum levels of d-serine, total serine, and d-serine/total serine ratios compared with healthy controls are shown in Table 3. The mean serum level of d-serine in patients with schizophrenia did not differ from that of healthy controls (*p* = 0.410; Mann–Whitney *U*-test). The mean total serine serum level was significantly lower (*p* = 0.006; Kolmogorov–Smirnov test) than that of healthy controls (Figure 1). The d-serine/total serine ratio was significantly higher in patients (*p* = 0.022; Kolmogorov–Smirnov test) than in healthy controls (Table 3). The d-serine (*r* = 0.19, *p* = 0.19; Spearman correlation coefficient) or total serine (*r* = 0.10, *p* = 0.47) serum levels were not associated with age in healthy controls.

**Table 2:** Population of the patients: demographical data, the results of clinical assessments, and amino acid serum levels (*n* = 50; males, *n* = 33; females, *n* = 17).

**Population of the patients**	**Mean ± SD**	**Median**
Age (years)	35.1 ± 11.0	31.5
Education (years)	13.1 ± 2.5	12.3
Illness duration (years)	10.4 ± 10.3	6
Dose of medication in chlorpromazine equivalents (mg)	344 ± 323	200
PANSS (total score)	49.1 ± 10.9	46.5
SANS (total score)	27.7 ± 18.5	24.5
D-Serine serum level (μmol/L)	3.3 ± 1.53	2.75
Total serine serum level (μmol/L)	83.1 ± 27.7	81.4
D-Serine/total serine ratio (%)	4.3 ± 2.3	3.6

*PANSS, the Positive and Negative Syndrome Scale; SANS, the Scale for the Assessment of Negative Symptoms*.

**Table 3:** Amino acid serum levels: comparison of patients and healthy controls.

		**Patients**	**Controls**	***p*-value**
		**(*n* = 50)**	**(*n* = 50)**	
D-Serine (μmol/L)	Mean ± SD	3.3 ± 1.53	3.1 ± 1.59	0.410^+^
	Median	2.75	2.73	
Total serine (μmol/L)	Mean ± SD	83.1 ± 27.7	100.9 ± 21.7	0.006^*^^++^
	Median	81.4	99.4	
D-Serine ratio to total serine	Mean ± SD	0.043 ± 0.023	0.031 ± 0.014	0.022^*^^++^
	Median	0.036	0.029	

+ *Mann–Whitney U-test*;

++ *Kolmogorov–Smirnov test*;

* *p < 0.05 was considered significant*.

We did not detect any significant association between the mean d-serine serum level and the mean scores of the total PANSS (*r* = 0.01, *p* = 0.93; Spearman correlation coefficient) or the total SANS (*r* = –0.08, *p* = 0.58) in patients with schizophrenia. The mean scores of either subscale of the PANSS or the SANS were not correlated with the d-serine serum levels (Table 4). However, we found a significant inverse correlation between the mean total serine serum level and the mean scores of the PANSS negative symptom subscale (*r* = –0.39, *p* = 0.005), the total SANS (*r* = –0.44, *p* = 0.001) (Figure 2), the SANS affective flattening or blunting subscale (*r* = –0.38, *p* = 0.006), the SANS alogia subscale (*r* = –0.37, *p* = 0.008), and the SANS anhedonia–asociality subscale (*r* = –0.52, *p* = 0.00009) (Table 4). We found a significant association between the mean d-serine/total serine ratio and the mean scores of the PANSS negative symptom subscale (*r* = 0.28, *p* = 0.049; Spearman correlation coefficient) but not with the total PANSS (*r* = 0.21, *p* = 0.15), total SANS (*r* = 0.25, *p* = 0.08), or other subscale mean scores of the PANSS and the SANS. The serum levels of d-serine or total serine were not related to age, illness duration, or chlorpromazine dose equivalents in patients (Table 4).

**Table 4:** The correlations between variables and amino acid serum levels in patients (*n* = 50).

**Variable**	**D-serine**		**Total serine**	
	***r***	***p-*value**	***r***	***p-*value**
Age	−0.07	0.64	−0.20	0.16
Illness duration	0.13	0.36	−0.14	0.33
Dose of medication in chlorpromazine equivalents	0.23	0.10	0.19	0.19
PANSS total score	0.01	0.93	−0.22	0.12
PANSS: P1–P7 score	0.08	0.60	0.14	0.34
PANSS: N1–N7 score	0.02	0.89	−0.39	0.005^*^
PANSS: G1–G16 score	0.01	0.94	−0.17	0.25
SANS total score	−0.08	0.58	−0.44	0.001^*^
SANS: affective flattening or blunting score	−0.003	0.98	−0.38	0.006^*^
SANS: alogia score	−0.16	0.27	−0.37	0.008^*^
SANS: avolition–apathy score	−0.04	0.76	−0.25	0.08
SANS: anhedonia–asociality score	−0.15	0.29	−0.52	0.00009^*^
SANS: attention score	0.07	0.65	−0.26	0.07

*PANSS, Positive and Negative Syndrome Scale; SANS, Scale for the Assessment of Negative Symptoms; P1–P7, positive symptom subscale; N1–N7, negative symptom subscale; G1–G16, general psychopathology subscale; r, Spearman correlation coefficient*;

* *p < 0.05 was considered significant*.

The neuropsychological tests grouped into executive functions domains were administered in all patients. Two patients did not finish the WCST. The average *Z* scores (sum of SDs from the mean) were determined from the results of the particular neuropsychological tests (Table 5). Performance in the TMT-A, the TMT-B, and in the TMT-B/TMT-A ratio did not correlate with the d-serine serum level or the d-serine/total serine ratio in patients with schizophrenia (Table 5). The ROCFT: reproduction after 3-min score was positively correlated with the d-serine serum level (*r* = 0.392, *p* < 0.05; Spearman correlation coefficient) and d-serine/total serine ratio (*r* = 0.280, *p* < 0.05). Similarly, the ROCFT: 30-min delayed reproduction score was positively correlated with the d-serine serum level (*r* = 0.421, *p* < 0.05), but not significantly with the d-serine/total serine ratio (*r* = 0.273, *p* = 0.055). The number of finished categories and percent of perseveration errors in the WCST did not correlate with d-serine serum level or d-serine/total serine ratio (Table 5). There were only non-significant differences of d-serine serum level or d-serine/total serine ratio in patients with the worst performance compared with the patients with the best performance when divided into the quartile groups according to the performance in the WCST (Table 6).

**Table 5:** The average *Z* scores from the particular neuropsychological tests, summary index 1 (TMT-B, ROCFT, and WCST) and summary index 2 (ROCFT, WCST) correlations with D-serine serum levels and D-serine/total serine ratio in patients with schizophrenia (*n* = 50).

**Neuropsychological test**	**The average *Z* score**	**D-serine serum level**		**D-serine/total serine ratio**	
		***r***	***p*-value**	***r***	***p*-value**
TMT-A (time)	−2.30	−0.207	0.150	−0.032	0.827
TMT-B (time)	−2.79	0.043	0.769	0.091	0.531
TMT-B/TMT-A (ratio)	—	−0.221	0.123	−0.050	0.732
ROCFT Copy (score)	−2.23	0.238	0.096	0.219	0.127
ROCFT reproduction after 3 min (score)	−0.94	0.392	0.005^*^	0.280	0.049^*^
ROCFT 30-min delayed reproduction (score)	−0.90	0.421	0.002^*^	0.273	0.055
WCST finished categories (quantity)	−0.49	−0.012	0.937	−0.015	0.922
WCST perseveration errors (%)	−0.30	0.125	0.399	0.107	0.469
Summary index 1 [TMT-B, ROCFT, WCST]	—	0.266	0.062	0.292	0.039^*^
Summary index 2 [ROCFT, WCST]	—	0.311	0.028^*^	0.336	0.017^*^

*TMT-A, Trail Making Test—time: part A; TMT-B, Trail Making Test—time: part B; TMT-B/TMT-A, Trail Making Test—time: part B/time: part A ratio; ROCFT, Rey–Osterrieth Complex Figure Test; WCST, Wisconsin Card Sorting Test; r, Spearman correlation coefficient*;

* *p < 0.05 was considered significant*.

**Table 6:** D-Serine serum levels and D-serine/total serine ratio—comparison of first and fourth quartiles of patients' population (*n* = 50) divided into groups according to performance in the WCST.

	**First quartile - the worst performance**	**Fourth quartile - the best performance**	***p*-value**
Finished categories (quantity)	Categories ≤ 2; *n* = 13	6 Categories; *n* = 22	
D-Serine serum level (mean ± SD; μmol/L)	2.9 ± 0.95	3.4 ± 1.8	0.48
D-Serine/total serine ratio (mean ± SD; %)	3.9 ± 2.0	4.2 ± 2.3	0.80
Perseveration errors (%)	19% ≤ errors; *n* = 13	Errors ≤ 9%; *n* = 16	
D-Serine serum level (mean ± SD; μmol/L)	2.8 ± 1.1	3.3 ± 1.3	0.40
D-Serine/total serine ratio (mean ± SD; %)	4.0 ± 2.0	4.3 ± 2.1	0.68

*WCST, Wisconsin Card Sorting Test; Mann–Whitney U-test; p < 0.05 was considered as significant*.

The summary index of the average *Z* scores from the TMT-B, the ROCFT and the WCST (summary index 1) insignificantly positively correlated with d-serine serum level (*r* = 0.266, *p* = 0.062; Spearman correlation coefficient), but significantly with d-serine/total serine ratio (*r* = 0.292, *p* < 0.05) (Table 5). Moreover, the summary index of the average *Z* scores from the ROCFT and the WCST (summary index 2) was significantly positively correlated with the d-serine serum level (*r* = 0.311, *p* < 0.05) or with d-serine/total serine ratio (*r* = 0.336, *p* < 0.05) in patients. We have found significant positive association of summary index 1 with the length of education (*r* = 0.451, *p* < 0.05; Spearman correlation coefficient), but no association with the SANS total score (*r* = –0.117, *p* = 0.419), the PANSS negative symptom subscale (*r* = –0.268, *p* = 0.059), age (*r* = –0.181, *p* = 0.208), or illness duration (*r* = –0.240, *p* = 0.093). Similar result for summary index 2 correlated to the length of education only was confirmed (*r* = 0.357, *p* < 0.05; Spearman correlation coefficient). The serum levels of d-serine and the d-serine/total serine ratio were also not related to the age, the length of education, or illness duration in patients with schizophrenia.

Significantly lower average serum level of d-serine and lower d-serine/total serine ratio (*p* < 0.05; Mann–Whitney *U*-test) were found in patients with the worst performance compared with the patients with the best performance when divided into the quartile groups according to the performance in the executive functions testing for summary index 2 (Table 7, Figure 3). However, we did not confirm the difference between the groups of patients when summary index 1 of executive functions testing was used. No differences were also found for summary index 1 or 2 in the average total serine serum level (*p* > 0.05) between the groups of patients (Table 7). There were also no differences in age, length of education, illness duration, dose of medication in chlorpromazine equivalents, or intensity of schizophrenic symptoms assessed by the PANSS or SANS scores between the groups of patients with the best or worst performance in executive functions testing.

**Table 7:** Amino acid serum levels—differences between the first and fourth quartiles of the patients' population (*n* = 50) divided into groups according to performance in executive functions testing, summary index 2.

	**First quartile - the best performance**	**Fourth quartile - the worst performance**	***p*-value**
	**(−1.1 SD ≤; *n* = 12)**	**(≤-8.8 SD; *n* = 12)**	
D-Serine serum level (mean ± SD; μmol/L)	3.51 ± 1.31	2.54 ± 0.59	0.049^*^
Total serine serum level (mean ± SD; μmol/L)	85.90 ± 31.09	91.00 ± 27.90	0.707
D-Serine/total serine ratio (mean ± SD; %)	4.64 ± 2.30	2.96 ± 0.82	0.030^*^

*Mann–Whitney U-test*;

* *p < 0.05 was considered as significant*.

## Discussion

The findings of lower d-serine levels in those suffering from the disease with more significant impairment of executive functions were consistent with our hypothesis that the extent of the function itself was associated with glutamatergic neurotransmission dysregulation in schizophrenia. Modifications in glutamatergic synapse function and structure point toward a typical underlying pathological aspect where several symptomatically unique cognition disorders are involved. The degree of impairment is linked negatively with the d-serine/total serine ratio in the sample being used within our study—it indicates modification of serine metabolism that is partially the reason behind NMDA reception breakdown and thus responsible for the pathogenesis of cognitive deficit in schizophrenia.

Cognitive deficit is considered an important dimension of schizophrenia, and it underlies other symptoms and affects the success of treatment, social adaptation ability, and patient quality of life. The depth of cognitive impairment has substantial interindividual variability. Harvey distinguishes slight damage corresponding to a reduction of 0.5 to 1 SD from the mean of healthy individuals, moderate damage to ~1.1 to 2 SD from the mean of healthy individuals, and severe cognitive impairment compared with 2 SD from the average (45). Gold (46) provides arguments supporting the hypothesis that the rates of cognitive deficits and psychopathology are relatively independent of the variable development trends of these two domains. Cognitive dysfunction is rather independent of positive schizophrenic symptoms, but there are studies finding an association between neurocognitive deficits and negative symptoms (47). A differential relationship between negative and cognitive symptoms in different stages of illness according to its duration was found in patients with schizophrenia (48). An association between impaired executive functions and the severity of negative symptoms was demonstrated in patients with a first episode of psychosis (49).

We did not confirm the association between schizophrenia and decreased d-serine serum levels (25, 26). In contrast, the total serine serum levels in our patients were lower, whereas the d-serine/total serine ratio was higher than that in healthy controls. We found that lower total serine serum levels were associated with a higher intensity of negative symptoms as assessed by the PANSS or the SANS. However, the intensity of negative symptoms was not correlated with the degree of impairment of executive functions in the sample of patients with stabilized, mostly chronic schizophrenia in our study. We confirmed the association between the level of executive functioning and the d-serine serum level or d-serine/total serine ratio, but no association of the d-serine serum level or d-serine/total serine ratio with the intensity of negative symptoms. The serum levels of d-serine or the d-serine/total serine ratios were not related to age, education, or illness duration in the subjects of our study. Similarly, the level of executive functioning was not related to age or illness duration, but was related only to the length of education in patients.

Our data suggest a possible implication of altered serine metabolism and NMDA receptor dysfunction in the pathogenesis of cognitive deficit and negative symptoms in schizophrenia. However, the interpretation of the results is limited by the size of the patients' population in our study. Data analysis was conducted as exploratory in order to identify potentially significant relationships between many under the condition of the rather small sample size and the relatively large number of compared parameters. The validation study on a larger sample is needed for inductive conclusions.

The role of serine metabolism in the glutamatergic dysfunction associated with schizophrenia requires further specification. Additional studies of EAAs in the periphery as well as in CSF may help to clarify the role of NMDA receptor dysfunction. Serum assays of endogenous d-serine may become a biomarker for its role at the NMDA receptor in the brain and eventually treatment sensitivity to exogenous EAAs in the future. However, the transport mechanisms of EAAs across the blood–brain barrier, the length, and the preceding medication will have to be considered. This study assumes that clinical and biochemical examinations of glutamatergic functional level could help split the disease into particular subtypes or categories. Finding the dysfunctionality linked to laboratory evidence changes in EAA metabolism enables a better treatment response, which in turn impacts the glutamatergic dysfunctional system.

## Ethics Statement

The study protocol was approved by the Local Ethics Committee (University Hospital Hradec Kralove Ethic Committee). The patients/participants provided their written informed consent to participate in this study.

## Author Contributions

JH developed the study, penned the protocol, gathered the data, and created the draft for the manuscript. RZ administered a neuropsychological battery and collected data. MV conducted the processes to determine EAA serum levels. BK, JM, MV, PD, and KK were instrumental in helping develop the manuscript draft and made considerable additions to the data interpretation and analysis. All authors contributed to the article and approved the submitted version.

## Conflict of Interest

The authors declare that the research was conducted in the absence of any commercial or financial relationships that could be construed as a potential conflict of interest.
